# Impact of the Volume and Distribution of Training Datasets in the Development of Deep-Learning Models for the Diagnosis of Colorectal Polyps in Endoscopy Images

**DOI:** 10.3390/jpm12091361

**Published:** 2022-08-24

**Authors:** Eun Jeong Gong, Chang Seok Bang, Jae Jun Lee, Young Joo Yang, Gwang Ho Baik

**Affiliations:** 1 Department of Internal Medicine, Hallym University College of Medicine, Chuncheon 24253, Korea; 2 Institute of New Frontier Research, Hallym University College of Medicine, Chuncheon 24253, Korea; 3 Department of Anesthesiology and Pain Medicine, Hallym University College of Medicine, Chuncheon 24253, Korea

**Keywords:** artificial intelligence, no code, endoscopy, colonoscopy, colonic neoplasms

## Abstract

Background: Establishment of an artificial intelligence model in gastrointestinal endoscopy has no standardized dataset. The optimal volume or class distribution of training datasets has not been evaluated. An artificial intelligence model was previously created by the authors to classify endoscopic images of colorectal polyps into four categories, including advanced colorectal cancer, early cancers/high-grade dysplasia, tubular adenoma, and nonneoplasm. The aim of this study was to evaluate the impact of the volume and distribution of training dataset classes in the development of deep-learning models for colorectal polyp histopathology prediction from endoscopic images. Methods: The same 3828 endoscopic images that were used to create earlier models were used. An additional 6838 images were used to find the optimal volume and class distribution for a deep-learning model. Various amounts of data volume and class distributions were tried to establish deep-learning models. The training of deep-learning models uniformly used no-code platform Neuro-T. Accuracy was the primary outcome on four-class prediction. Results: The highest internal-test classification accuracy in the original dataset, doubled dataset, and tripled dataset was commonly shown by doubling the proportion of data for fewer categories (2:2:1:1 for advanced colorectal cancer: early cancers/high-grade dysplasia: tubular adenoma: non-neoplasm). Doubling the proportion of data for fewer categories in the original dataset showed the highest accuracy (86.4%, 95% confidence interval: 85.0–97.8%) compared to that of the doubled or tripled dataset. The total required number of images in this performance was only 2418 images. Gradient-weighted class activation mapping confirmed that the part that the deep-learning model pays attention to coincides with the part that the endoscopist pays attention to. Conclusion: As a result of a data-volume-dependent performance plateau in the classification model of colonoscopy, a dataset that has been doubled or tripled is not always beneficial to training. Deep-learning models would be more accurate if the proportion of fewer category lesions was increased.

## 1. Introduction

Gastroenterology has applied artificial intelligence (AI) in terms of computer vision or machine learning analysis [1,2]. Various image interpretation models of endoscopy or ultrasound have been developed in the context of computer vision [3,4]. It would save a significant amount of time and effort on the part of medical professionals in the field if medical procedures could be automated with the help of AI. They can devote more of their time to gathering more resources and achieving the best possible outcomes for patients [3].

Endoscopists typically remove all colorectal polyps identified during screening colonoscopies since this approach has been shown to limit the progression of adenoma-carcinoma sequence [2,5,6]. However, since the removal of an adenoma is linked to cancer prevention, it may be cost-effective to distinguish it from a hyperplastic polyp [2]. Methods for reliable prediction of polyp histology based on visual evaluation of gross morphology are not always accurate and adenoma detection rates are known to diminish with an increasing practitioner workload [6,7]. As an alternative to visual inspection, artificial intelligence diagnosis utilizing deep learning makes it possible to automatically recognize, classify, and segment images with high accuracy [1,4,6].

In order to predict the histology of colorectal polyps from 3828 endoscopic images, the authors developed a deep-learning model. This model successfully predicted the histology of four different lesion classes, including advanced colorectal cancer (ACC), early cancers/high-grade dysplasia (ECC/HGD), tubular adenoma (TA) with or without low-grade dysplasia (LGD), and nonneoplasm, with a 67.3 percent internal-test accuracy and 79.2 percent external-test accuracy [8]. A relatively small number of images were available in the input training data. Moreover, the proportion of ACC and ECC/HGD was relatively small compared to TA or nonneoplasm [9].

Establishment of the AI model in gastrointestinal endoscopy has no standardized dataset. The optimal volume or class distribution of training datasets has not been evaluated [3,4]. As a result, the purpose of this study was to assess the impact of the training dataset volume and distribution on the development of deep-learning models for the prediction of colorectal polyp histology from white-light endoscopy images.

## 2. Methods

### 2.1. Input Datasets

By creating and assessing deep-learning models with no-code tools with varying levels of data volume and class distributions, this study expands on a prior study [8,9]. The new deep-learning models were constructed using the same 3828 white-light endoscopic pictures as input for diagnostic performance comparison. An additional 6838 images were used to find the optimal volume and class distributions for the deep-learning model.

The class distribution of ACC and ECC/HGD was lower than that of TA and nonneoplasm in the original dataset. Differentiation of ECC/HGD and TA was not accurate compared to other categories in previous model establishment [8,9]. Therefore, various distributions were tested, such as the 1:1:1:1 for ACC, ECC/HGD, TA, and nonneoplasm or doubling the number of fewer categories (ACC and ECC/HGD) or doubling the less accurate categories (ACC and TA). Additionally, various amounts of data volume tried to establish deep-learning models (Table 1).

Input training data process collection was previously described [8,9]. In brief, subjects diagnosed and treated for colorectal lesions at three university hospitals (Chuncheon Sacred Heart, Dong-tan Sacred Heart, and Hallym University Sacred Heart Hospital) were identified retrospectively between 2008 and 2017, and pathologically confirmed colonoscopy images were collected in JPEG format with a minimum resolution of 640,480 pixels [8,9]. An additional 6838 images collected between 2018 and April 2022 from Chuncheon Sacred Heart Hospital were used for the experiment to find the optimal volume or class distribution. The distribution of additional 6838 images are as follows: 546 ACCs, 189 ECC/HGDs, 3586 TAs, and 2517 nonneoplasms.

Performance verification (external test) was conducted using 3818 novel images from consecutive patients receiving colonoscopy between 2017 and 2021 at four university hospitals (Chuncheon Sacred Heart Hospital, Kangdong Sacred Heart Hospital, Inje University Ilsan Paik Hospital, and Gangneung Asan Hospital). All images used for validation (included in the external-test datasets) were different from those used for training [9] (Table 2).

### 2.2. Labeling of the Training Dataset

**Figure 1:**
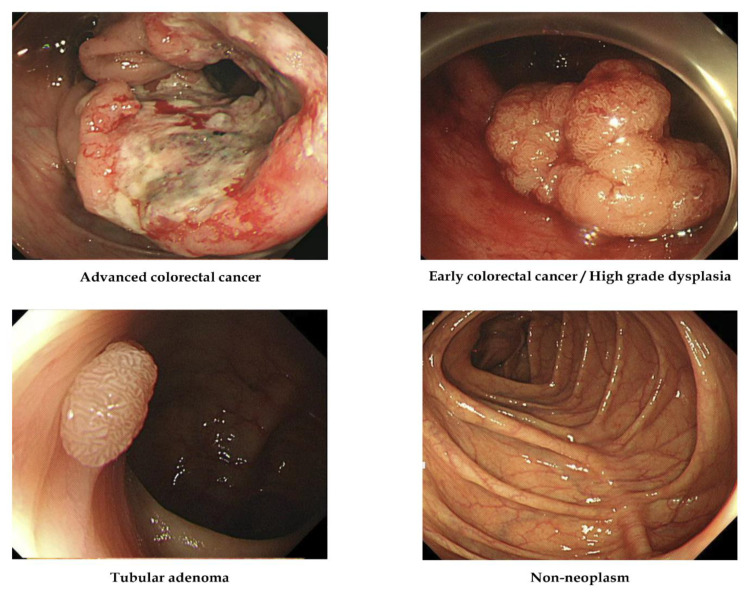
Representative images in each category used to establish artificial intelligence models. Representative examples of lesion images in each category are shown.

Following endoscopic or surgical removal, all images were labeled based on pathological evaluation. Histologically, lesions were classified into one of the four categories listed below [8,9]: (1) adenocarcinoma; (2) TA with HGD (in situ or intramucosal cancer); (3) TA with or without LGD; and (4) hyperplastic polyp, inflammatory polyp, lymphoid polyp, leiomyoma, lipoma, or another nonneoplastic lesion. The clinical stage, including the invasion depth, determined the therapeutic strategy, such as surgery or endoscopic removal, so lesions were classified into four alternative classes: (1) ACC (stages T2, T3, and T4 cancers), (2) ECC/HGD (stage T1 cancers and HGD), (3) TA, and (4) nonneoplasm. There was no image that was included in more than one pathological class (i.e., all were mutually exclusive). Figure 1 demonstrates representative images [8,9].

### 2.3. Establishment of an Artificial Intelligence Model

Training of artificial intelligence models uniformly used no-code platform Neuro-T (version 2.3.2, Neurocle Inc., Seoul, Korea). This tool creates convolutional neural network-based deep-learning models for lesion detection or classification tasks by analyzing the features of the dataset and self-discovering optimal hyperparameters [8,9,10].

### 2.4. Training and Data Preprocessing

This study aimed to find the optimal volume or class distribution for a colorectal lesion classification model. Therefore, a common preprocessing and hyperparameter optimizing tool was used. This study’s no-code deep-learning tool has unique automated preprocessing functions and training options. This function performs image resizing transformations on input images. To identify the best performing deep-learning models, all images were resized to 512 × 480 pixels before training and on-premise software-based model establishment with automated hyperparameter optimization.

As a default option, Neuro-T software was used to input training images randomly divided into training and internal-test sets at a 9:1 ratio. Table 2 describes each training and internal-test dataset. The model training hardware consisted of four RTX 2080 Ti GPUs, dual Xeon CPUs, and 256 GB RAM.

### 2.5. Primary Outcome and Statistics

The internal-test accuracy was the primary outcome. The precision or positive predictive value (defined as (true positive/true positive + false positive)), recall or sensitivity (defined as (true positive/true positive + false negative)), and F1 score (2 precision recall/precision + recall) were additional performance metrics. Chuncheon Sacred Heart Hospital’s Institutional Review Board (2018-05) approved this study.

## 3. Results

### 3.1. Diagnostic Performances of the Deep-Learning Models According to Various Data Volume and Class Distributions

**Figure 2:**
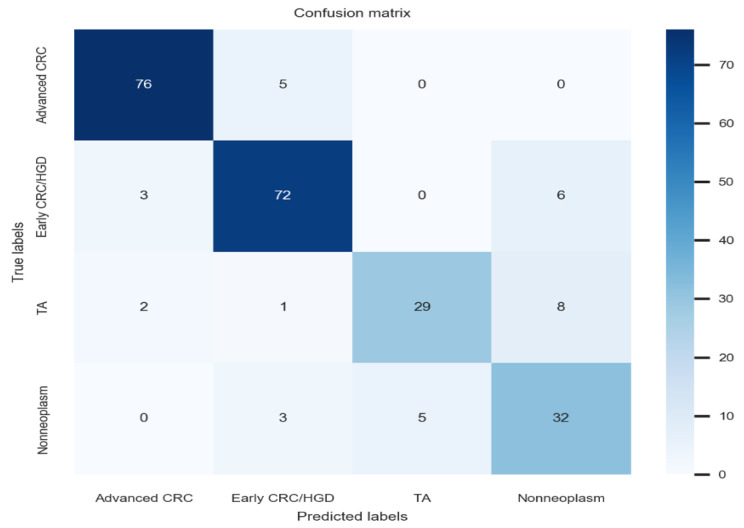
Best-performing artificial intelligence model confusion matrix (internal-test). ACC: advanced colorectal cancer, ECC/HGD: early cancers/high-grade dysplasia, TA: tubular adenoma.

Various amounts of data volume and class distributions tried to establish deep-learning models. Doubling the proportion of data for fewer categories (2:2:1:1 for ACC:ECC/HGD:TA:nonneoplasm) commonly showed the highest internal-test classification accuracy in the original dataset, doubled dataset, and tripled dataset. Doubling the proportion of data for fewer categories in the original dataset showed the highest accuracy (86.4%, 95% confidence interval: 85.0–97.8%) compared to that of the doubled or tripled datasets (precision: 84.4%, recall: 83.8%, F1 score: 84.1%) (Table 3). The total required number of images in this performance was only 2418 images. Figure 2 demonstrates the confusion matrix for the deep-learning model with the best performance. The hyperparameters used in the establishment of the best-performing model were as follows: Resnet-based neural network. A. Batch Size: 56, B. Epoch: 95, C. Number of Layers: 18, D. Optimizer: adam, E. Initial Learning Rate: 0.00146.

### 3.2. Gradient-Weighted Class Activation Mapping

**Figure 3:**
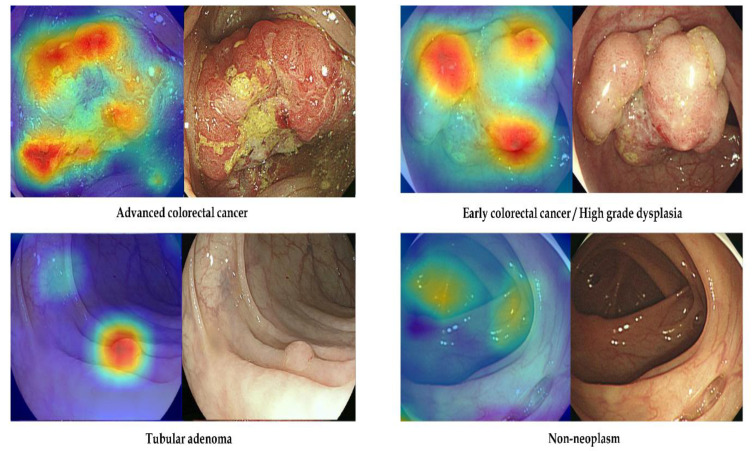
Representative examples of correctly classified lesions in the internal-test datasets. Left: gradient-weighted class activation mapping image. Right: white-light endoscopic image.

The gradient-weighted class activation mapping function of the no-code tool utilized in this work demonstrates the discriminative properties employed by the established model for classification. Figure 3 displays representative samples from the internal tests with the right classifications made by the model in place. The gradient-weighted class activation map in Figure 3 reveals that the established models’ discrimination features, such as the surface mucosal irregularity, color changes, and protruded regions were similar to those used by endoscopists during visual inspection [11].

## 4. Discussion

This study demonstrated that increasing the proportion of data for fewer categories in the training dataset showed an improved internal-test classification accuracy. Doubling the proportion of data for fewer categories in the original dataset showed the highest accuracy (86.4%). The total required number of images in this performance was only 2418 images. A large amount of data is generally preferred in the establishment of a deep-learning classification model; however, presumably because of a data-volume-dependent performance plateau, this study showed that it is not always beneficial to training [12,13].

The quality or quantity of the baseline training data influences the performance of the deep-learning model [14]. We do not, however, have qualified quality indicators for the training data. High-quality representative data reflecting real-world practice should be collected to avoid spectrum bias (data imbalance) or overfitting (modeling error, which occurs when a certain learning model is excessively tailored to the training dataset and predictions are not well generalized to new datasets) [2,15]. There have been many studies to establish a gastrointestinal endoscopy deep-learning model [1,4]. However, data-centric AI studies are lacking in the field of gastrointestinal endoscopy [16].

Depending on the quality, nature, or characteristics of the data, AI models are trained using data, and AI models generate predictions. We require certified data that reflects a real-world problem. Data from multiple institutions would be preferable over data from a single institution. The class distribution of data is also crucial. The formation of biased models would be the result of a class imbalance [17]. We can use undersampling of the majority classes or oversampling of the minority classes to solve this problem. The inherent pitfall of selection bias should be recognized for the collection of input data.

How much data is needed to reasonably approximate the unknown underlying mapping function in deep learning is unknown in the context of the amount of training data [15,18,19]. Too little training data would generally result in poor approximation. A large amount of data is conversely not necessarily good for training [1]. A data-volume-dependent performance plateau occurs, which is related to whether the data has sufficient features and complexity of the background model [18]. The training time might be too long if there is too much data.

This study confirmed that increasing the proportion of data for fewer categories is associated with improved accuracy, especially for doubling the proportion in the training dataset, and a doubled or tripled amount of data is not always beneficial to training as a result of a data-volume-dependent performance plateau. A deep-learning model can be created by anyone who can organize data. There is, however, no universal rule for this. As a result, when it comes to colon neoplasia diagnostic models, the preparation of data based on the findings of this study is advantageous. The current colonoscopy polyp image dataset contains 590 to 1000 images [20,21]. Despite the difficulty of experimenting with a large dataset in such a situation, the authors investigated how to create a deep-learning model with an optimal performance using as much data as possible.

There have been several inevitable limitations. First, there is no study on the impact of datasets’ quality in the development of a deep-learning model in the colonoscopy classification model. There is no available baseline quality in our dataset, although we collected only clear and easily recognizable images. Therefore, this might influence the classification performance, irrespective of the class distribution or the amount of data. Second, performance verification with an external test was not done. Although an established model might be optimal for in-hospital usage, the generalization possibility of the performance was not confirmed in this study. We are planning to reestablish deep-learning classification model in the colonoscopy with data for multi-institution and an increased proportion of fewer category lesions in a future study because the aim of this study was revealing a proof-of-concept. Third, the primary outcome was the classification accuracy in this study. However, accuracy might not be the best performance metric in this class-imbalanced dataset [22]. Although other performance metrics, such as the precision, recall, and F1 score, commonly showed substantial value in the highest performance model, comprehensive interpretation of the performance combined with various performance metrics is still important. Fourth, the purpose of this study was not about the amount and distribution of datasets that are generally applicable to all deep-learning models. Since we only focused on the colon neoplasia diagnosis model, the results of this study are limited to the topic of this study.

In conclusion, a dataset that has been doubled or tripled is not always beneficial to training as a result of a data-volume-dependent performance plateau in the classification model of colonoscopy. Deep-learning models would be more accurate if the proportion of fewer category lesions was increased.

## Figures and Tables

**Table 1:** Histological class distribution in input datasets.

	Original Dataset	Even Distribution of Each Class	Doubling Data for Fewer Categories	Doubling Data for Less Accurate Categories	Doubling the Number of Total Data; (Original Dataset of 3828 Images with New 3964 Images)	Even Distribution of Each Class	Doubling Data for Fewer Categories	Doubling Data for Less Accurate Categories	Tripling the Number of Total Data; (Original Dataset of 3828 Images with New 6838 Images)	Even Distribution of Each Class	Doubling Data for Fewer Categories	Doubling Data for Less Accurate Categories
Overall	3828	3224	2418	2418	7792	3540	2656	2656	10,666	3980	2986	2986
Advanced colorectal cancer	810	806	806	403	994	885	885	443	1356	995	995	498
Early colorectal cancer/high-grade dysplasia	806	806	806	806	885	885	885	885	995	995	995	995
Tubular adenoma with or without low-grade dysplasia	1316	806	403	806	3634	885	443	885	4902	995	498	995
Nonneoplasm	896	806	403	403	2279	885	443	443	3413	995	498	498

The number of images adjusted for the amount of data and the ratio for each class is described in the table.

**Table 2:** Training and internal-test dataset distribution in each input dataset.

	Original Dataset	Even Distribution of Each Class	Doubling Data for Fewer Categories	Doubling Data for Less Accurate Categories	Doubling the Number of Total Data	Even Distribution of Each Class	Doubling Data for Fewer Categories	Doubling Data for Less Accurate Categories	Tripling the Number of Total Data	Even Distribution of Each Class	Doubling Data for Fewer Categories	Doubling Data for Less Accurate Categories
Overall	3828	3224	2418	2418	7792	3540	2656	2656	10,666	3980	2986	2986
Training dataset	3444	2900	2176	2176	7013	3184	2258	2258	9599	3582	3688	3688
Internal-test dataset	384	324	242	242	779	356	398	398	1067	398	298	298

The number of images adjusted for the amount of data and the ratio for each class divided by training and internal-test data are described in the table.

**Table 3:** Internal-test accuracy according to each data volume and class distribution.

Data Distribution (ACC: ECC/HGD: TA: Nonneoplasm)	Original Dataset (*n* = 3828)	Doubling the Total Data; Combined Dataset (*n* = 7792)	Tripling the Total Data; (*n* = 10,666)
Raw data	75.3%	67.5%	72.4%
Even distribution (1:1:1:1)	72.8% (*n* = 3224)	75.6% (*n* = 3540)	74.0% (*n* = 3980)
Doubling the proportion of data for fewer categories (2:2:1:1)	86.4% (*n* = 2418)	78.9% (*n* = 2656)	82.4% (*n* = 2986)
Doubling the proportion of data for less accurate categories (1:2:2:1)	81.5% (*n* = 2418)	74.9% (*n* = 2656)	79.2% (*n* = 2986)

ACC: advanced colorectal cancer, ECC/HGD: early cancers/high-grade dysplasia, TA: tubular adenoma. The internal-test accuracy according to the amount of data and the ratio for each class is described in the table.

## Data Availability

All data are available from the corresponding author upon reasonable request.

## Abbreviations

ACC	advanced colorectal cancer
ECC/HGD	early cancers/high-grade dysplasia
TA	tubular adenoma
LGD	low-grade dysplasia
